# Suspected Drug-Induced Liver Injury Associated With Ceftriaxone: A Case Report

**DOI:** 10.7759/cureus.88419

**Published:** 2025-07-21

**Authors:** Allison D Zettwoch, Okelue E Okobi, Mercedes Millan, Angelica Perez Fonte, Raphael S Figueroa, Tolulope A Fatuki, Raul J De la Vega, Sergio Hernandez Borges, Miguel Diaz-Miret

**Affiliations:** 1 Family Medicine, Larkin Community Hospital Palm Springs Campus, Hialeah, USA; 2 Medicine, Nova Southeastern University Dr. Kiran C. Patel College of Osteopathic Medicine, Miami, USA; 3 Internal Medicine, Larkin Community Hospital Palm Springs Campus, Hialeah, USA; 4 Internal Medicine, Veterans Affairs Medical Center, Grand Junction, USA; 5 Internal Medicine/Family Medicine, Larkin Community Hospital Palm Springs Campus, Hialeah, USA

**Keywords:** ceftriaxone, drug-induced liver injury, elderly patient, geriatric pharmacology, hepatotoxicity

## Abstract

An 85-year-old female patient with multiple comorbidities, including Parkinson's disease, hyperthyroidism, congestive heart failure, and Alzheimer's, presented with dyspnea, weakness, and cough. She was found to have new-onset atrial fibrillation and a left frontal lobe infarct. Initially treated with levofloxacin and then azithromycin, she later developed right lung infiltrates, and ceftriaxone 2g IV daily was initiated. Within 48 hours, liver enzymes aspartate aminotransferase (AST) and alanine transaminase (ALT) rose markedly (from 11/26 to 452/415 U/L). We promptly discontinued ceftriaxone in response and considered alternative antibiotics. The multidisciplinary team opted for supportive management with daily liver function monitoring, resulting in gradual normalization of transaminases. This case highlights the importance of prompt drug discontinuation, use of structured drug-induced liver injury (DILI) assessment tools, and adherence to geriatric prescribing guidelines.

## Introduction

Drugs are a major cause of liver injury, accounting for almost half of all acute liver failure cases, and drug-induced liver injury (DILI) may result in severe hepatitis, mixed pattern, and cholestasis [1-3]. Several drugs have been linked to comparatively high to very low DILI rates, and among the most common classes are statins, acetaminophen products, antibiotics, and non-steroidal anti-inflammatory drugs (NSAIDs) [2,3]. DILI represents a considerable clinical issue, with antibiotics identified as one of the contributing factors. DILI also spans diverse clinical manifestations that vary from mild biochemical abnormalities to severe liver failure. Ceftriaxone, a third-generation cephalosporin, is widely used for its broad-spectrum antibacterial efficacy and advantageous safety profile, including its longer half-life, higher tissue penetration rate, better safety profile, and wider spectrum [4-6]. Nevertheless, similar to other cephalosporins with reduced hepatotoxicity, there are fewer documented cases of ceftriaxone-induced hepatotoxicity, although they are infrequent [1-3]. Particularly, hepatotoxicity linked to antibiotics is often asymptomatic and is normally characterized by milder and unrecognized hepatic injury. Diagnosis is fundamentally clinical, given that no biomarkers or diagnostic tests are available, and needs exclusion of other potential causes responsible for abnormal liver tests [4,5]. To establish the potential for a drug to be responsible for adverse reactions, physicians often use the Naranjo’s algorithm alongside the Roussel Uclaf Causality Assessment Method (RUCAM), which is a score system, to assess the clinical, serological, radiologic, and biochemical features of liver damage, showing the probability of a particular treatment causing hepatic injury [6,7]. Therefore, this report discusses a case of acute liver injury following ceftriaxone administration in an elderly female patient, highlighting the importance of awareness and monitoring for such adverse effects.

## Case presentation

**Figure 1:**
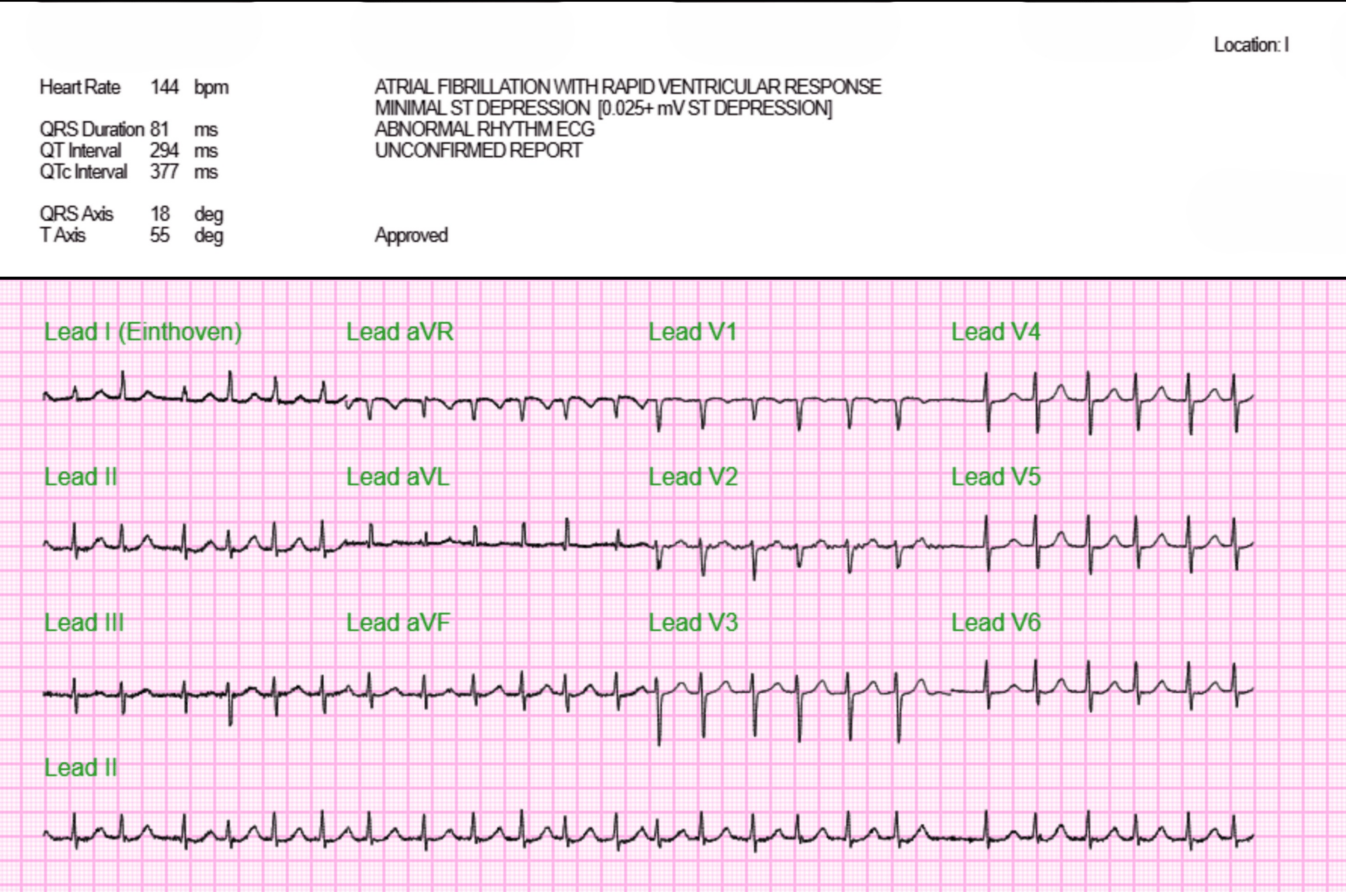
Initial EKG with new onset atrial fibrillation

An 85-year-old female patient with Parkinson's disease, hyperthyroidism, anxiety, congestive heart failure, and Alzheimer's disease presented with generalized weakness, shortness of breath, and a productive cough of three days. The patient was oriented only to self. Her niece provided collateral history. Vitals showed mild tachypnea and an oxygen saturation of 92% on room air. Lung examination revealed bilateral wheezing. The cardiovascular exam was notable for new-onset atrial fibrillation confirmed via electrocardiogram (EKG) (Figure 1). Baseline liver function tests drawn two weeks prior to this admission were within normal limits: aspartate aminotransferase (AST) 11 U/L and alanine transaminase (ALT) 26 U/L.

**Figure 2:**
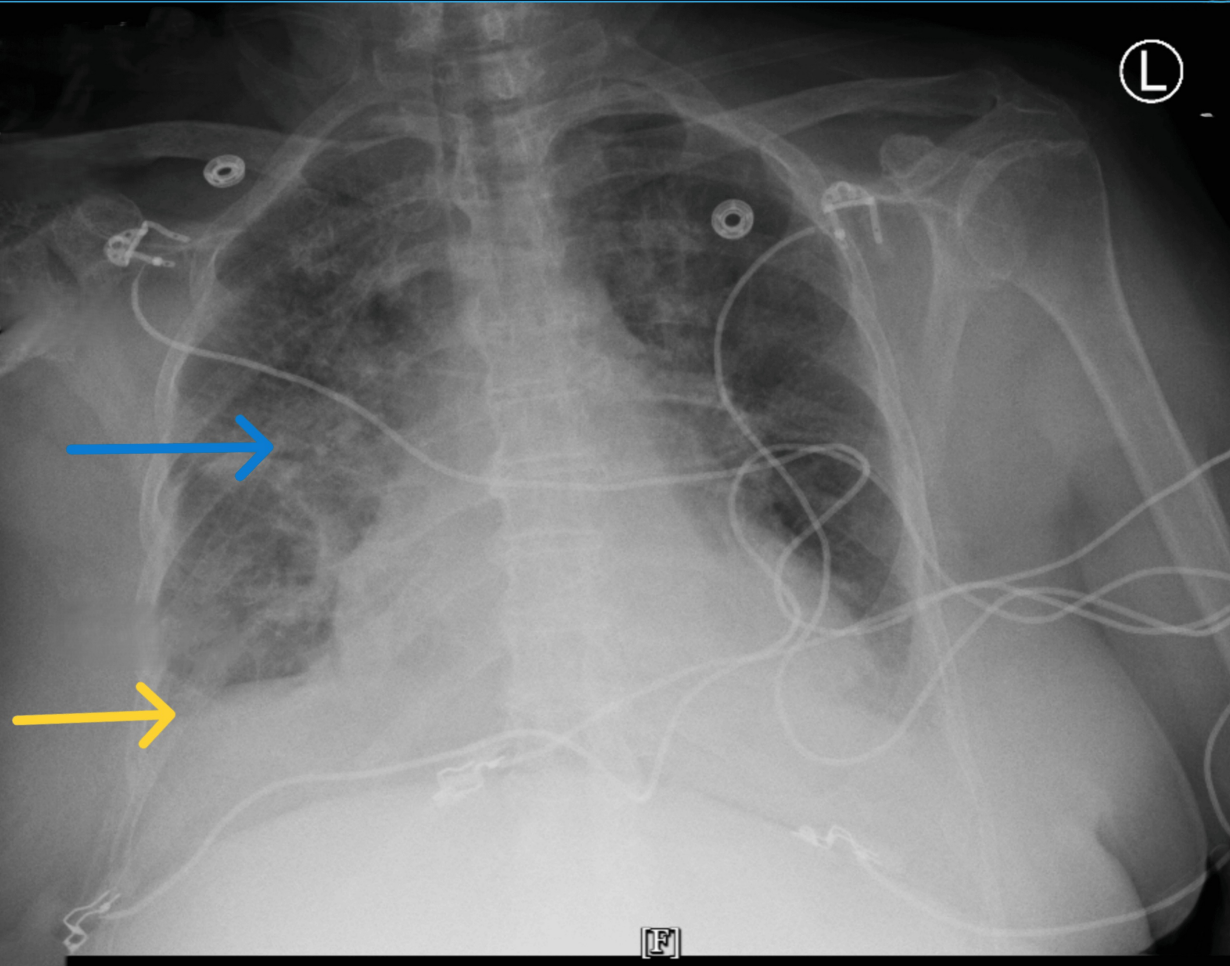
Features suggestive of chest infection Blue arrow pointing at right lung opacities are favored to represent an infectious/inflammatory process, with blunting of the right costophrenic angle (yellow arrow), which may represent a small right pleural effusion versus a summation of tissue.

Chest imaging revealed changes consistent with chest infection (Figure 2).

**Figure 3:**
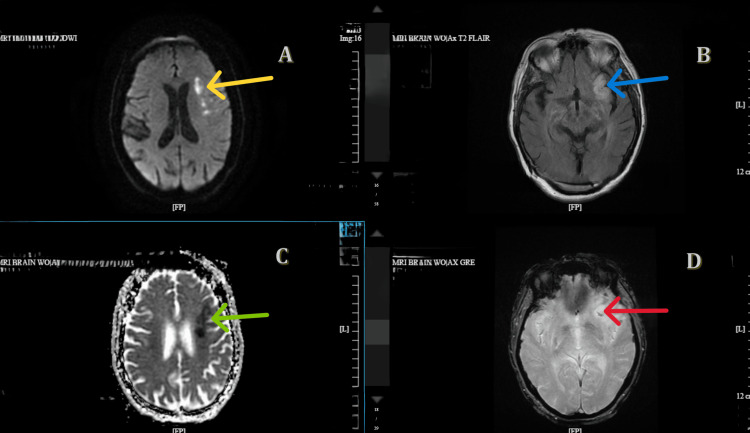
Findings concerning for acute ischemic infarct/stroke to early subacute ischemic infarct/stroke in left frontal lobe, with hemorrhagic transformation MRI of the brain showing acute ischemic infarct hyperintensity on DWI (yellow arrow in panel A), FLAIR (blue arrow in panel B), hypointensity on ADC (green arrow in panel C), and hemorrhagic changes (red arrow in panel D). DWI: diffusion-weighted imaging; FLAIR: fluid-attenuated inversion recovery; ADC: apparent diffusion coefficient

Brain magnetic resonance imaging (MRI) revealed a left frontal ischemic infarct (Figure 3), correlating with her acute speech changes.

**Figure 4:**
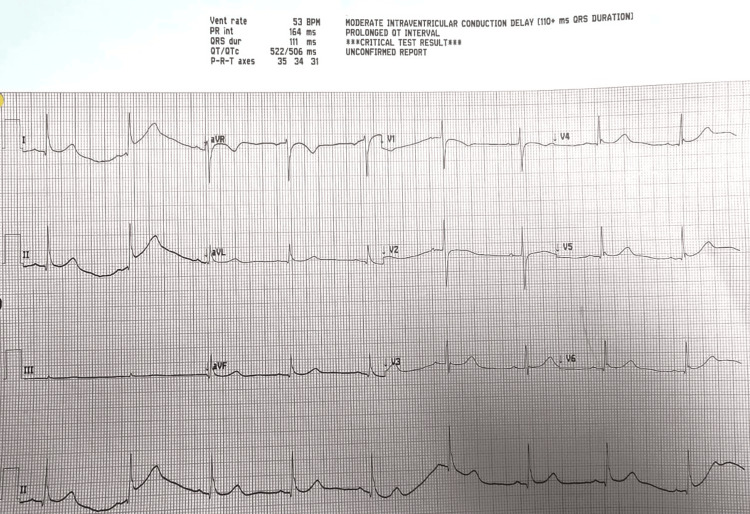
EKG showing features of prolonged QTc QTc: corrected QT interval

The patient's chest infection was initially managed with levofloxacin and later switched to azithromycin due to corrected QT interval (QTc) concerns (Figure 4).

**Figure 5:**
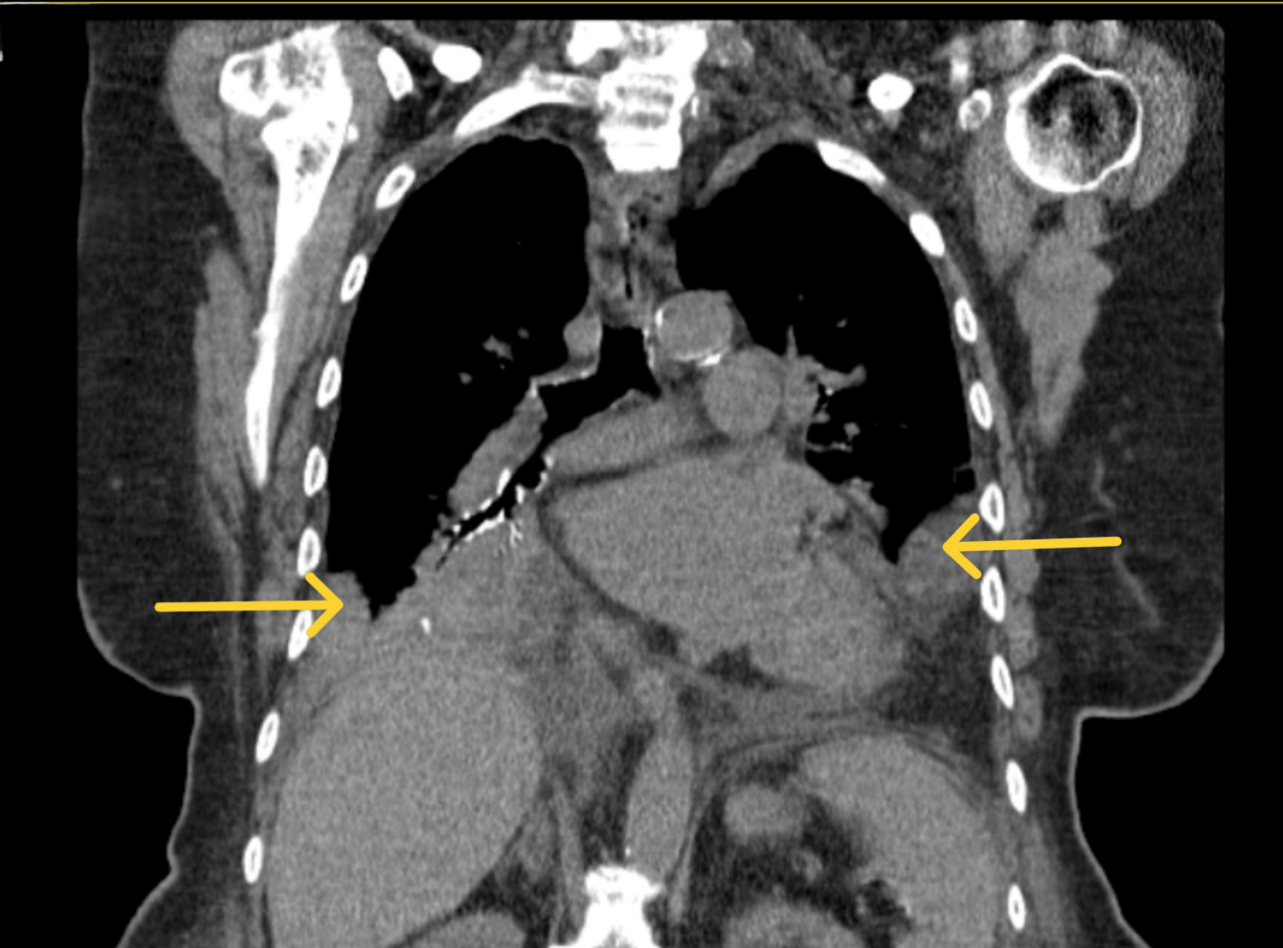
CT chest showing bilateral pleural effusions (R > L) with adjacent RLL possibly consistent with pneumonic process and likely parapneumonic effusion, correlate clinically Yellow arrows show signs of parapneumonic effusion. RLL: right lower limb; R: right; L: left

In the intensive care unit (ICU), the patient was started on a low-dose amiodarone drip and aspirin. Anticoagulation with enoxaparin was withheld due to features of a recent stroke seen on brain MRI. On hospital day five, the patient deteriorated clinically with worsening right-sided opacities on imaging, suggestive of a worsening pneumonic process on repeat imaging (Figure 5).

The patient was empirically started on ceftriaxone 2 g IV daily due to clinical indication. Liver function tests (LFTs) obtained on hospital Day 1 (prior to ceftriaxone administration) showed AST 44 U/L and ALT 180 U/L (Table 1). Within 48 hours of starting ceftriaxone, AST rose to 452 U/L, ALT to 415 U/L, gamma-glutamyl transferase (GGT) to 115 U/L, and alkaline phosphatase (ALP) was mildly elevated; total bilirubin and international normalized ratio (INR) remained normal. These findings sparked concerns about drug-induced hepatotoxicity, prompting us to promptly discontinue ceftriaxone following the Day 2 LFT results. A formal DILI evaluation using the RUCAM yielded a score indicating "probable" causality. Amiodarone was considered a co-contributor but was continued with close monitoring due to its critical role in the management of atrial fibrillation (Afib). The team considered switching to cefotaxime as a safer cephalosporin alternative. Gastroenterology recommended daily LFT monitoring and abdominal ultrasound to assess biliary involvement (which was unremarkable).

**Table 1:** Patient laboratory results AST (U/L): aspartate aminotransferase (units per liter); ALT (U/L): alanine aminotransferase (units per liter); ALP (U/L): alkaline phosphatase (units per liter); GGT (U/L): gamma-glutamyl transferase (units per liter); TBILI: total bilirubin; INR: international normalized ratio; BNP (pg/mL): B-type natriuretic peptide (picograms per milliliter); HbA1c: Hemoglobin A1c; Na (mmol/L): sodium concentration in millimoles per liter; K (mmol/L): potassium concentration in millimoles per liter; Cl (mmol/L): chloride concentration in millimoles per liter; A/G ratio: albumin/globulin ratio; Hep A: hepatitis A; Hep B core Ab: Hepatitis B core antibody; HBsAg: Hepatitis B surface antigen; Hep C Ab: Hep C core antibody; BUN/creatinine ratio: blood urea nitrogen to creatinine ratio; Hep A IgM: Hepatitis A immunoglobulin M; Hep B surface Ab: Hepatitis B surface antibody

Lab parameter (unit)	Normal limit	Day 1	Day 2	Day 3	Day 4	Day 5	Day 6	Day 7	Day 8	Day 9	Day 10
AST (U/L)	15-46	44	43	34	44	43	250	452	300	150	134
ALT (U/L)	0-50	180	116	98	180	116	250	415	310	200	167
ALP (U/L)	38-126	112	104	96	112	104	110	120	104	96	89
GGT (U/L)	8-610	-	-	-	-	-	85	115	100	90	56
TBILI (mg/dL)	0.2-1.3	0.9	0.9	0.8	0.9	0.9	0.9	0.9	0.9	0.8	0.8
INR	0.8-1.2	1.0	1.0	1.0	1.0	1.0	1.0	1.0	1.0	1.0	1.1
BNP (pg/mL)	0-450	2940	2940	2940	2940	2940	-	-	-	-	-
HbA1c (%)	4.0-5.6	6.3	-	-	-	-	-	-	-	-	-
Na (mmol/L)	137-145	139	139	140	139	139	139	139	139	140	141
K (mmol/L)	3.5-5.1	4.2	3.8	3.8	4.2	3.8	4.1	4.0	4.2	3.9	3.7
Cl (mmol/L)	98-107	105	101	101	105	101	102	101	100	102	103
Protein (g/dL)	6.3-8.2	6.6	6.5	6.4	6.6	6.5	6.5	6.4	6.5	6.4	6.4
Albumin (g/dL)	3.5-5.0	3.7	3.7	3.7	3.7	3.7	3.7	3.7	3.7	3.7	3.6
Globulin (g/dL)	2.3-3.5	2.9	2.8	2.7	2.9	2.8	2.8	2.7	2.8	2.7	2.8
A/G ratio	1.5-2.5	1.3 L	1.3 L	1.4 L	1.3 L	1.3 L	1.4 L	1.4 L	1.4 L	1.4 L	-
Hep A	-	Non-reactive	-	-	-	Non-reactive	-	-	-	-	-
Hep B core Ab	-	Non-reactive	-	-	-	Non-reactive	-	-	-	Non-reactive	-
HBsAg	-	Non-reactive	-	-	-	Non-reactive	-	-	-	Non-reactive	-
Hep C Ab	-	Non-reactive	-	-	-	Non-reactive	-	-	-	Non-reactive	-
Creatinine (mg/dL)	0.52-1.04	1.41	1.49	1.53	1.41	1.49	1.4	1.38	1.35	1.32	1.31
BUN/creatinine ratio	5-20	24	23	25	24	23	21	20	19	18	18
Hep A IgM	-	Non-reactive	-	-	-	Non-reactive	-	-	-	Non-reactive	-
Hep B surface Ab	-	Non-reactive	-	-	-	Non-reactive	-	-	-	Non-reactive	-

The patient was monitored closely with no development of jaundice, encephalopathy, or INR derangement. Liver enzymes peaked post-ceftriaxone but began trending down after discontinuation (Table 1). She was clinically stable, and her pneumonia symptoms improved with cefotaxime. The patient’s other medical issues, including Afib, hypothyroidism, and ischemic stroke, remained stable with regular monitoring and adjustments as needed.

**Figure 6:**
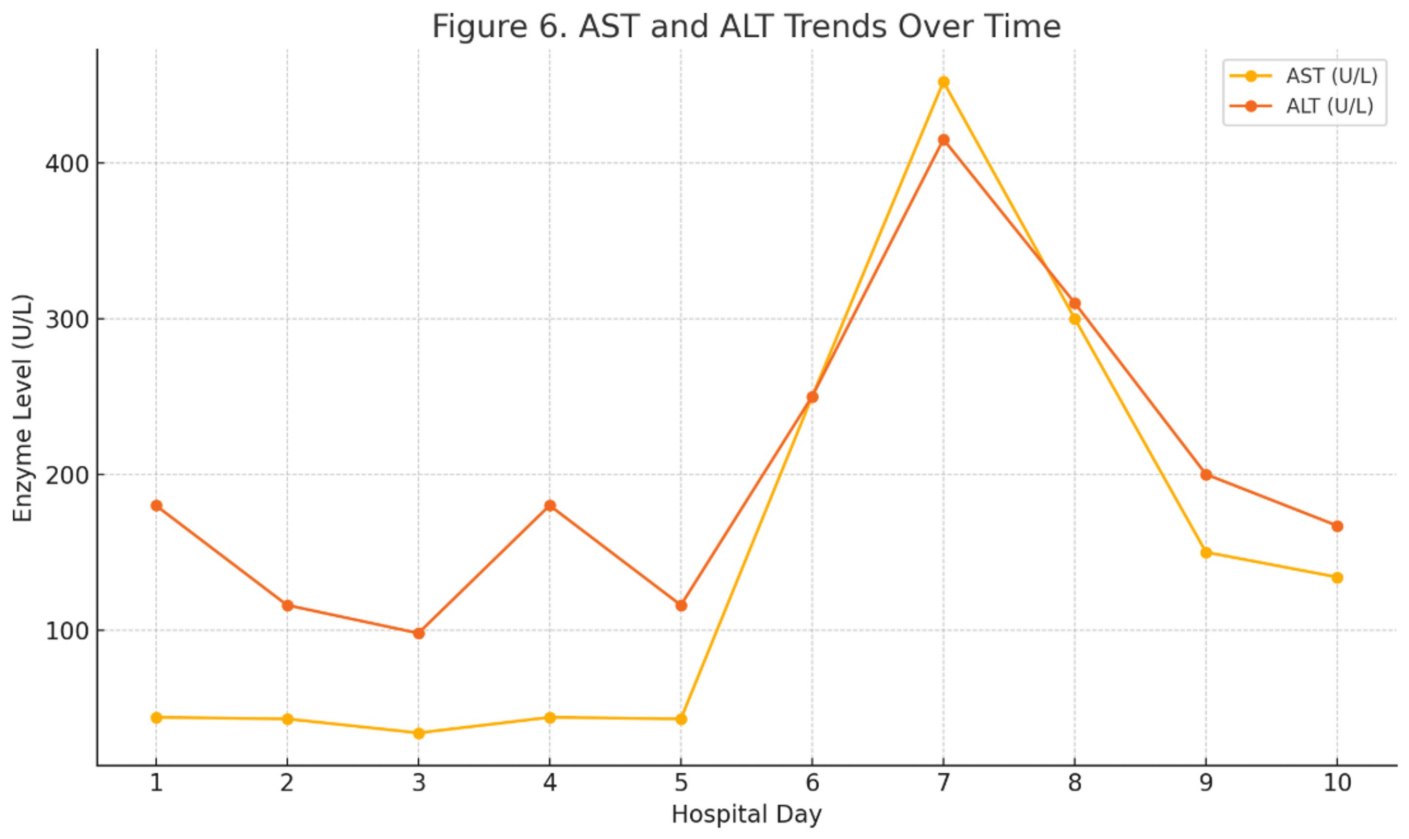
Line graph depicting AST and ALT trends from Day 1 through Day 10, illustrating the temporal association between ceftriaxone administration (Day 1–2) and enzyme elevation followed by normalization post-discontinuation Days 1–2 represent the period of ceftriaxone administration; a clear spike in both enzymes is observed on Day 7, followed by a gradual decline after discontinuation and switch to cefotaxime. AST: aspartate aminotransferase; ALT: alanine transaminase

Figure 6 illustrates the dynamic changes in serum transaminases over the first 10 days of hospitalization, highlighting the temporal relationship with ceftriaxone administration and its discontinuation. As shown, AST and ALT remained near baseline on Days 1-2 (prior to and during the initial ceftriaxone dose), then rose sharply, peaking on Day 7 (AST 452 U/L; ALT 415 U/L), before trending downward following the switch to cefotaxime. This graphical representation underscores the rapid enzyme elevation within 48 hours of ceftriaxone exposure and the subsequent recovery of hepatic function once the drug was withdrawn.

## Discussion

This case exemplifies the importance of vigilance when prescribing ceftriaxone in older adults. This case shows the potential for ceftriaxone to induce liver injury, even at standard doses, particularly in vulnerable populations. Although hepatotoxicity is rare at standard doses, factors such as advanced age, polypharmacy (notably amiodarone), and renal impairment increased her risk. The rapid elevation of liver enzymes following ceftriaxone administration aligns with findings from other reports. A retrospective cohort study by Nakaharai et al. revealed that high-dose ceftriaxone (≥4 g/day) was associated with a significantly higher incidence of liver injury compared to standard doses [1]. Specifically, 16.2% of patients receiving high-dose ceftriaxone experienced liver injury versus 2.1% on standard doses [1]. Although our patient received a standard dose, her advanced age and multiple comorbidities may have increased her susceptibility. Individual case reports further illustrate ceftriaxone-induced hepatotoxicity. Peker et al. described a 12-year-old boy who developed toxic hepatitis after ceftriaxone treatment for tonsillitis, with AST and ALT levels reaching 819 U/L and 871 U/L, respectively [2]. Discontinuation of ceftriaxone and administration of methylprednisolone led to the normalization of liver enzymes [2]. In a similar case, Guarino et al. reported a 77-year-old woman who got sudden liver inflammation after taking ceftriaxone for pneumonia, with her AST and ALT levels reaching 11,961 U/L and 6,111 U/L, respectively [3]. In our patient, ceftriaxone was discontinued on Day 2 and replaced with cefotaxime; close monitoring and a multidisciplinary approach were crucial in her recovery, preventing further hepatic injury. Stopping ceftriaxone and providing supportive care helped her improve. The patient's clinical course, characterized by significant enzyme elevation with minimal clinical symptoms, reflects patterns observed in previously reported cases.

Mechanism of action, metabolism, and hepatotoxic potential of ceftriaxone

Ceftriaxone is a third-generation cephalosporin with broad-spectrum activity against Gram-positive and Gram-negative bacteria. Its bactericidal effect stems from its ability to inhibit bacterial cell wall synthesis. Specifically, ceftriaxone binds to and inactivates penicillin-binding proteins (PBPs), enzymes crucial for peptidoglycan cross-linking in bacterial cell walls. This disruption weakens the cell wall, leading to osmotic instability and eventual bacterial lysis. Due to its stability against many beta-lactamases, ceftriaxone is particularly effective against resistant bacterial strains [8].

Metabolism and the role of the liver

Unlike many antibiotics, ceftriaxone is primarily eliminated by dual routes; 50-60% is excreted unchanged in the urine via the kidneys, while 40-50% is excreted in bile and feces via hepatic processing [8]. Importantly, ceftriaxone undergoes minimal hepatic metabolism, as it is not extensively transformed into metabolites by liver enzymes [9]. However, its significant biliary excretion involves active transport processes in hepatocytes, which may contribute to hepatobiliary complications [10]. The liver’s role in ceftriaxone elimination becomes relevant under certain circumstances. High ceftriaxone concentrations in bile can precipitate insoluble complexes, sometimes forming biliary sludge or stones. These conditions can trigger local inflammation, cholestasis, or hepatic injury, particularly in predisposed individuals [11].

Risk or confounding factors

Although liver injury caused by ceftriaxone is uncommon, certain factors can increase an individual's susceptibility to it. Firstly, there is a risk of dose-dependent hepatotoxicity. Despite receiving 2 g of ceftriaxone, our patient's DILI has implicated dose-dependent toxicity. High doses of ceftriaxone, typically ≥4 g/day, are more likely to result in liver enzyme elevation. These effects are attributed to dose-related saturation of biliary excretion pathways, which leads to the accumulation of toxic intermediates [8,11]. Secondly, unlike in our patient, underlying hepatic conditions have also been an exacerbating factor. Patients with pre-existing liver disease, such as non-alcoholic fatty liver disease (NAFLD) or viral hepatitis, have a compromised ability to handle the hepatobiliary burden of ceftriaxone [10]. Thirdly, just like in this patient, age-related vulnerability has also been implicated. Older adults, like the patient in this case, may experience reduced hepatic and renal clearance, increasing drug exposure and risk of toxicity [8,12]. Fourth, reports have identified polypharmacy and drug interactions as potential factors. Concomitant use of drugs metabolized by similar pathways can exacerbate liver injury by overloading the hepatobiliary excretion system [9]. This pattern was typically seen in this patient with concomitant amiodarone use. Other factors include immune-mediated mechanisms. Hypersensitivity reactions to ceftriaxone may cause immune-mediated hepatotoxicity. This process involves the formation of drug-protein adducts, which trigger an autoimmune-like attack on hepatocytes [9,10]. Another potential factor is a congestive or obstructive hepatic condition. In some patients, ceftriaxone induces biliary sludge or gallstones, leading to obstruction and secondary cholestasis. This condition can indirectly cause liver injury by impairing bile flow [11,12].

Defining liver failure and comparing it with the patient's case

Liver failure, as defined by the American College of Gastroenterology (ACG), is the loss of liver function that occurs either acutely (over days to weeks) or in a chronic context (chronic liver disease progressing to decompensation) [13]. Acute liver failure (ALF) is characterized by the rapid onset of severe liver dysfunction, typically manifesting as coagulopathy (INR ≥ 1.5) and hepatic encephalopathy in patients without pre-existing liver disease [14]. Chronic liver failure typically follows cirrhosis and is marked by complications such as portal hypertension, ascites, and progressive hepatic dysfunction [14]. For a diagnosis of ALF, key criteria include significant elevations in liver enzymes (ALT and AST), prolonged prothrombin time (PT/INR ≥1.5), and evidence of encephalopathy resulting from hepatic dysfunction. Bilirubin levels are often elevated, indicating impaired bilirubin conjugation and excretion. In patients with pre-existing liver disease, acute-on-chronic liver failure (ACLF) may occur and includes criteria of multi-organ failure precipitated by an acute insult [15]. In our patient's case, while liver enzymes were markedly elevated following ceftriaxone administration (ALT 415 IU/L and AST 452 IU/L), these values alone do not confirm liver failure. The patient’s INR and bilirubin levels were not reported as abnormal, nor was there evidence of hepatic encephalopathy or signs of decompensation such as jaundice, ascites, or coagulopathy. Furthermore, the patient maintained stable systemic function without multi-organ involvement, which would be required to meet the criteria for ACLF. The absence of prolonged PT/INR or encephalopathy indicates that the patient’s liver dysfunction, while significant, does not meet the ACG criteria for liver failure. The hepatotoxicity observed appears to be an isolated drug-induced liver injury (DILI), which in this case was associated with ceftriaxone administration. With appropriate monitoring and clinical management, the patient’s liver function can potentially return to baseline without progressing to liver failure.

Differential diagnosis

Potential causes of acute transaminase elevation were explored. Viral hepatitis panels (HAV, HBV, HCV) were negative, ruling out viral reactivation. Ischemic hepatitis was unlikely given stable hemodynamics without hypotensive episodes. Autoimmune hepatitis markers were unremarkable, and abdominal ultrasound showed no biliary obstruction or cholestatic changes. These findings support drug-induced liver injury as the most probable etiology.

Limitations

The present study was a single case report, which limits the generalizability of the findings to broader populations, age groups, or clinical contexts, and the existence of other comorbidities and polypharmacy may have been a confounder in this case. Also, genetic susceptibility or underlying metabolic enzyme polymorphisms (e.g., uridine diphosphate-glucuronosyltransferases, cytochrome P450 variants) were not explored. Although an ultrasound was done, there were no further imaging or more sensitive modalities to explore other possible causes of hepatobiliary pathologies.

Learning points

First, always consider DILI in elderly patients on ceftriaxone. Secondly, discontinue the suspected offending drug early when safe alternatives exist. Third, utilize structured tools like RUCAM for causality. Additionally, monitor LFTs regularly and involve multidisciplinary teams early. Lastly, align care with geriatric pharmacologic guidelines to minimize iatrogenic risks.

## Conclusions

Ceftriaxone-induced liver injury, while rare, should be part of the differential diagnosis in elderly patients with rising liver enzymes. Prompt discontinuation, structured causality assessment, daily monitoring, and guideline-driven substitutions form the cornerstone of effective management. This case adds to the growing body of evidence advocating cautious antibiotic use in geriatric populations.
